# Trends in recombinant protein use in animal production

**DOI:** 10.1186/s12934-017-0654-4

**Published:** 2017-03-04

**Authors:** Laia Gifre, Anna Arís, Àlex Bach, Elena Garcia-Fruitós

**Affiliations:** 1 Department of Ruminant Production, Institut de Recerca i Tecnologia Agroalimentàries (IRTA), 08140 Caldes de Montbui, Spain; 2 Institució Catalana de Recerca i Estudis Avançats (ICREA), Barcelona, Spain

**Keywords:** Recombinant proteins, Animal production, Recombinant expression systems, Reproductive hormones, Fibrolytic enzymes, Therapeutic molecules

## Abstract

Recombinant technologies have made possible the production of a broad catalogue of proteins of interest, including those used for animal production. The most widely studied proteins for the animal sector are those with an important role in reproduction, feed efficiency, and health. Nowadays, mammalian cells and fungi are the preferred choice for recombinant production of hormones for reproductive purposes and fibrolytic enzymes to enhance animal performance, respectively. However, the development of low-cost products is a priority, particularly in livestock. The study of cell factories such as yeast and bacteria has notably increased in the last decades to make the new developed reproductive hormones and fibrolytic enzymes a real alternative to the marketed ones. Important efforts have also been invested to developing new recombinant strategies for prevention and therapy, including passive immunization and modulation of the immune system. This offers the possibility to reduce the use of antibiotics by controlling physiological processes and improve the efficacy of preventing infections. Thus, nowadays different recombinant fibrolytic enzymes, hormones, and therapeutic molecules with optimized properties have been successfully produced through cost-effective processes using microbial cell factories. However, despite the important achievements for reducing protein production expenses, alternative strategies to further reduce these costs are still required. In this context, it is necessary to make a giant leap towards the use of novel strategies, such as nanotechnology, that combined with recombinant technology would make recombinant molecules affordable for animal industry.

## Background

Unquestionably, the production of recombinant proteins has become a reality thanks to the discovery of the recombinant DNA technology in the seventies. The implementation of this technology has made the production of most protein of interest recombinantly possible. Before this, proteins of interest were extracted from their natural sources through expensive processes and poor yields [1]. However, nowadays, scientists can routinely isolate or synthesize genes and clone them in a suitable expression system for production purposes at industrial scale. Although there is a wide range of cell factories that are currently used for recombinant protein production purposes, including bacteria, yeast, fungi, algae, insect cells, and mammalian cells [2], the bacterium *Escherichia coli* has become the workhorse in this field. This is not only due to the low production costs associated to this prokaryotic expression system, but also to the number of available tools that makes this process easy to implement. The first functional recombinant protein (somatostatin) was produced in 1977 using *E. coli* as cell host [3] and, just some years later, Genentech Inc. launched a recombinant human insulin also produced in *E. coli*.

However, despite the undeniable advances made in the recombinant protein production field, production processes, and more importantly downstream product processing, have important associated costs. This is particularly limiting for the production of recombinant proteins for animal science, where the development of low-cost products and strategies are a priority.

Despite the existence of some limitations, the use of recombinant proteins in animal science has clearly increased in the last decades. Looking at the overall bibliography where recombinant proteins are being used, it comes out that one of the most studied field is the endocrine system [4–8]. Indeed, there are already some commercial recombinant hormones available and many groups are working on their implementation to improve reproduction of livestock. Many research studies also focus on less-demanded proteins that need specific customization of production procedures according to particular features. Recombinant engineered proteins are being widely explored for the design of both prophylactic treatments and therapeutic strategies. Also, several enzymes are being recombinantly produced with the aim of improving efficiency of feed conversion into edible products.

This article offers an overview of recombinant proteins produced in microbial cell factories, focusing in three fundamental pillars for animal production: (1) reproduction, (2) feed efficiency, and (3) health. This review seeks not only to draw a map of the current situation, but also to highlight the relevance that recombinant technologies could have in a near future for the animal sector. However, all the recombinant products involved in vaccination procedures have been excluded from this revision because they have been thoroughly covered in other articles and reviews [9–11].

## Recombinant hormones in reproduction

Animal reproduction is one of the areas where production of recombinant protein is broadly used [4, 12, 13]. Reproductive hormones have a critical role in the regulation of the male reproductive function, female reproductive cycle, and the maintenance of pregnancy in the dams. In animal production, these hormones are used for two opposite purposes: enhancing female fertility by regulating ovulation and/or facilitating embryo implantation, and on the other hand improving meat quality by sterilized males.

Follicle stimulating hormone (FSH) and luteinizing hormone (LH) are gonadotropins secreted by the anterior pituitary gland when induced by the gonadotropin releasing hormone (GnRH), secreted by the hypothalamus [14–16]. These glycoproteins, together with the chorionic gonadotropin (CG) secreted by the placenta of primates and equids, are used in animal breeding management for superovulation purposes in females, and to stimulate testosterone production and spermatogenesis in males. On the other hand, inhibin, which is secreted by both male and female gonads, has a great importance because it exerts a negative feedback to the anterior pituitary lowering the secretion of gonadotropin and thus their effects.

At present, the most commonly used hormones for reproductive purposes are purified from animal-derived material such as pituitaries. Despite being a widespread practice, it has significant associated problems such as: (i) batch-to-batch inconsistencies leading to variations in the superovulatory responses between animals [17, 18], (ii) purity problems because of the presence of other hormones contaminating the sample [16], and (iii) the possible contamination with disease-transmitting agents (for a more detailed review see [13]). All these require the development and application of cumbersome purification protocols to concentrate and purify the protein to guarantee the quality of the final product. In this context, production of recombinant hormones appears as an attractive solution to overcome these drawbacks, having reproducible superovulation effects [12] by using considerably smaller doses than those utilized with animal-derived hormones [19].

Gonadotropins are structured in non-covalent heterodimers, composed of a common α subunit and a hormone-specific β subunit. To obtain a functional hormone, both subunits must be assembled together [14, 20] posing an important bottleneck for its recombinant production. However, and because the interaction between subunits is not an absolute requirement for receptor activation ([21] and references therein), functional single-chain gonadotropin analogs have been successfully developed recombinantly by merging their α and β subunit genes in a single sequence [21–24]. Nevertheless, and even if the steroidogenic response is achieved, the structural differences in the hormone analogs commonly imply differences in steroid secreted levels [21]. Furthermore, the 2 subunits of gonadotropins are glycosylated after translation generating pools of different glycoforms (gonadotropin varieties) with different half-lives and activity efficiencies [25, 26]. Thus, since reproductive hormones require a N-glycosylation [20], recombinant production has been carried out mainly in mammalian or insect cells [13, 27]. However, the production of proteins in large amounts in these eukaryotic systems is expensive, difficult, and time-consuming. Furthermore, protein hormones are usually produced at low yields. Alternatively, yeast (eukaryotic microorganisms), and in some cases *E. coli*, are being explored as a cost-effective and easy-to-work systems. *Pichia pastoris* (reclassified as *Komagataella pastoris*) is the most commonly used yeast in this context, because it efficiently secretes the protein produced and adds N-glycosylations. Even though *P. pastoris* can glycosylate proteins, it should be stressed that only one specific strain described by Jacobs et al. is able to introduce a mammalian-type N-glycosylation [28].

### Follicle stimulating hormone (FSH)

FSH acts in ovaries in conjugation with LH. They are responsible of stimulating the granulose cells and promoting follicle growth preceding the ovulation stage. Also, both gonadotropins stimulate the dominant follicle to ovulate. In males, FSH is responsible for stimulating the Sertoli cells in testes for spermatogenesis, together with testosterone secreted by the action of LH in Leydig cells.

Administering exogenous FSH has been a typical practice for promoting superovulation and spermatogenesis in different animal species, and due to the disadvantages associated with pituitary-extracted hormones, recombinant hormone formulations started to rise. There are commercial forms of FSH derived from pituitary glands such as Folltropin-V (Bioniche Animal Health-now Vetoquinol-) and Pluset (Calier), which also contain LH (Table 1). The commercially-available recombinant FSH used for animal follicular development and superovulation has been mostly produced by Chinese hamster ovary (CHO) cells. Some examples are Follistim (follitropin beta; Merck Serono (USA) -now Merck-), Puregon (follitropin beta; Organon B.V. (Europe) -now merged with MSD-) and Gonal-F (follitropin alpha; Merck). Also, AspenBio Pharma (named Venaxis, Inc. since 2012) tooks a relevant role as a supplier of bovine and equine single-chain and long-acting FSH analogs (BoviPureFSH™ and EquiPureFSH™) (Table 1). In some cases, human embryonic kidney (HEK) cells have been chosen as a cell factory to produce bovine FSH (Nanocore Biotecnologia SA) used to supplement culture medium for in vitro follicle development in mares [29], dogs [30], goats, and sheep [31] (Table 1). The equine reproduction research industry has clearly been a user and promoter of this drug to stimulate follicular growth [6, 19, 32, 33] and ovulation [34] in mares. Even so, although the human version of this long-acting and single-chain FSH, Elonva (Corifollitropin α; MSD) was already available in 1992 [35–38], the bovine and equine analogs did not reach the market until 2008 [6].

**Table 1:** Marketed follicle stimulating hormone (FSH), Luteinizing hormone (LH) and chorionic gonadotropin (CG) for animal reproduction

Name	Cell factory/origin	Company
FSH		
Follistim^®^	CHO cells	Merck Serono (USA)-now Merck-
Puregon^®^	CHO cells	Organon B.V. (Europe) -now merged with MSD-
Gonal-F^®^	CHO cells	Merck
BoviPureFSH™	CHO cells	AspenBio Pharma (Venaxis, Inc. since 2012)
EquiPureFSH™	CHO cells	AspenBio Pharma (Venaxis, Inc. since 2012)
FSH	HEK cells	Nanocore Biotecnologia SA
Folltropin-V^®^	Pituitary gland	Bioniche Animal Health-now Vetoquinol-
Pluset^®^	Pituitary gland	Calier
LH		
BoviPureLH™	CHO cells	AspenBio Pharma (Venaxis, Inc. since 2012)
EquiPureLH™	CHO cells	AspenBio Pharma (Venaxis, Inc. since 2012)
Luveris^®^	CHO cells	Merck Serono (USA) -now Merck-
Pluset^®^	Pituitary gland	Calier
CG		
Pregnyl	Urine	Organon B.V. (Europe) -now merged with MSD-
Folligon^®^	Serum	MSD
Novormon^®^ 5000	Chorion	Syntex
PG600^®^	Chorion and serum	MSD

Although all commercial recombinant FSH are produced in mammalian cell lines and research is still being conducted in this area [39, 40], yeasts, which are relatively inexpensive and effective expression systems, are gaining importance in this field of study. The most used yeast to produce FSH is *K. pastoris.* Bovine, porcine, ovine, and primate recombinant FSH have been produced with *K. pastoris* in studies carried out to improve the yields with this affordable cell factory [41–44]. Although in these studies the activity was only tested in vitro, results indicated that proteins produced were functional and with a great potential to be applied in vivo. *K. pastoris* was also explored for the production of in vitro tested single-chain ovine FSH analogs [45], as well as for the production of fish FSH that showed the capacity to stimulate steroidogenesis and ovarian development in vivo [46–48]. Moreover, eel FSH produced in *K*. *pastoris* has been proven in vitro, fostering steroidogenesis in immature eel testis tissue [49] and spermatogenesis [50] (for more information about recombinant fish gonadotropin development, see [27]). Also, the yeast *Hansenula polymorpha* (*Pichia angusta*) has been used to express bovine FSH, which has been successfully tested in vivo in mice for follicular growth purposes [51].

During the last decades, different strategies to increase FSH production in recombinant yeast have been evaluated including the co-expression of a disulfide isomerase [52], the co-expression of *Saccharomyces cerevisiae*-derived calnexin [51] or codon usage optimization [51, 52]. Medium optimization has also been deeply studied to optimize cell densities and production yields [53]. Thus far, although the yields achieved using both *K. pastoris* and *P. angusta* have notably been improved, they are still insufficient to be used for commercial purposes and further research is necessary in this context. Interestingly, a non-glycosylated form of recombinant bovine FSH produced in a bacterial expression system (*E. coli*) showed to be able to stimulate ovarian development in rats [54], emerging as a promising alternative to be further explored.

### Luteinizing hormone (LH) and chorionic gonadotropin (CG)

In addition to stimulating ovulation in females, along with FSH, LH stimulates the following development of the corpus luteum. In males, LH is responsible for testosterone secretion in the testes by the Leydig cells, which in turn stimulate spermatogenesis in Sertoli cells. On the other hand, CG supports embryo implantation and pregnancy [20, 55]. In horses, both LH and CGβ subunit derive from the same gene, whereas in primates the two gonadotropins are derived from different genes although they share 80% of their amino acid sequence [55]. CGβ and LHβ subunits differ only in the length of their carboxyl terminal regions. The CG has a longer region because of a peptide called carboxyl terminal peptide (CTP) that provides the CG with more glycosylation places and prolong CG half-life by reducing its renal clearance [56]. These extra glycosylation sites have been fused to FSH [6, 19, 32–34] and LH [21, 24, 57–59], which do not naturally contain this sequence, to achieve long-acting hormones. This allowed the reduction in the number of injections for superovulation treatments [13, 36, 40]. Interestingly, given the similarities between the LH and CG, both hormones bind to the LH receptor.

Recombinant LH commercially-available used for animal reproduction purposes is also being produced in CHO cells (Table 1). As an example, the single-chain EquiPureLH (Venaxis) was used in mares in combination with EquiPureFSH for superovulation treatment [32], or in combination with a pituitary FSH (eFSH; Bioniche Animal Health) to study their effect in follicle and oocyte development [60]. Also, LH has been administered in mares as a model to treat the luteinizing unruptured follicle syndrome in humans [61]. Furthermore, the human-indicated Luveris (hLH; Merck) has been used for early embryonic development treatments in rabbits [62] combined with Gonal-F (see the FSH section), and in mice [63] combined with Pregnyl (hCG; Organon) (Table 1).

Both dimer- [64, 65] and single-chain forms [21, 24, 57–59, 66, 67] of LH and CG have been studied in CHO expression system. Also, human recombinant CG was obtained by CHO cell expression for the construction of a chimera hCG-boCTP used to study the potential of a CTP-like sequence present but not expressed in the β subunit of bovine (among other mammal species) LH [68].

Insect cells have been chosen as an alternative to the expensive CHO cells, for the recombinant equine LH and CG production [23, 69, 70] (for a review see [71]). Moreover, *K. pastoris* has been explored to produce recombinant human LH (hLH) and CG (hCG). Gupta et al. already described hCG production in 1999 [72] and some years later other authors successfully produced both hCG [73, 74] and hLH [74] in *K. pastoris*. Although the hLH expressed by *K. pastoris* showed to be less glycosylated and to have less affinity for the receptor than that naturally expressed in pituitary, it was fully active [74]. Gonadotropins from fish have also been produced in *K. pastoris*, showing the capacity to stimulate steroidogenesis in tilapia [27, 75]. *E. coli*-derived hCGβ was obtained for the first time in 1994 by Huth and coworkers [76]. Briefly, the β subunit of this hormone was recovered from purified and solubilized inclusion bodies (IBs), and refolded in vitro to conduct structural and biological studies. After dimerization with a urinary hCGα subunit, the resulting hormone activated ovulation in vivo in rats although its β subunit could not be glycosylated by *E. coli*. Thus, this study showed that it is possible to produce biologically-active hormones in a prokaryotic organism, which lacks the capacity to introduce post-translational modifications. In this context, Mukhopadhyay et al. also produced hCGβ in *E. coli* for vaccination purposes [77]. This, together with the fact that other hormones, such as growth hormones from different origins have been broadly studied in this recombinant system (buffalo [78], caprine [78], bovine [79], ovine [80] and porcine [81]), showing activities equivalent to those found in natural hormones, suggests that *E. coli* has a bold potential in this field. Although, thus far, mammalian cells have been the gonadotropin producers *per excellence* [13, 27], articles published show that microbes can be used as a real alternative for the production of biologically active CG and LH through economic and facile processes [36–39, 49, 67, 70].

### Inhibin

During the ovarian cycle, inhibin (which belongs to the TGFβ superfamily) is mainly secreted by the large developing follicles causing the atresia of the smaller ones [82, 83]. Its secretion in response to increasing levels of FSH in the gonads triggers a negative feedback to the anterior pituitary lowering FSH circulating levels. Therefore, inhibin regulates follicle development and ovulation rates in females, and spermatogenesis in males.

Yan et al. have extensively reviewed inhibin effects (and of its neutralization) on follicle and embryo development [84]. Superovulation treatments with exogenous gonadotropins result in increased numbers of developing follicles, which in turn lead to inhibin concentration rise in plasma [85] and a quantitatively and qualitatively reduced oocyte and embryo development [86]. Consequently, an immunization practice against inhibin, combined with a conventional superovulation protocol, has proven to enhance the quality of the resulting embryos both in vitro and in vivo [87–89]. This immunization has been achieved through the administration of exogenous inhibin leading to antibody production against this glycoprotein.

From a research perspective, recombinant inhibin or its α subunit used in a wide number of studies has been produced in bacteria, and more specifically in *E. coli* [90]. In this context, genetic engineering has been used to improve inhibin production in *E. coli* [91]. However, in many other cases inhibin has been produced in mammalian cells [16, 92]. Importantly, recombinant inhibin has been used as an antigen for the immunization against endogenous inhibin in hens [93, 94], cockerels [95], heifers [87, 89, 96], water buffaloes [88], guinea pigs [97], goats [98], and sheep [99–102]. Only in a minority of cases, the recombinant inhibin α subunit used is obtained synthetically [103–106] or purified from follicular fluid [107]. Thus, the production of inhibin, also known as the “superovulatory vaccine”, has been extensively studied in recombinant bacteria. Although currently inhibin has not been marketed, the research done with this hormone is promising.

## Recombinant fibrolytic enzymes

The efficiency of plant cell wall digestibility by endogenous enzymes in animals is low. Basically, most non-starch polysaccharides components present in the animal feed are indigestible by mammalian enzymes, which precludes a full recovery of the nutritional value of the diet. Furthermore, a fraction of the digestible nutrients (i.e., sugars, starch, fat, protein) becomes undigestible because are wrapped by non-starch polysaccharides [108, 109]. Thus supplementation of diets with exogenous enzymes to enhance animal performance has been a practice extensively used for decades to increase feed conversion rate (proportion of growth relative to the amount of feed consumed). Initially, fibrolytic enzymes were used essentially in non-ruminant animals (pigs and poultry), since it was believed that rumen proteases and ruminal microorganisms were able to efficiently degrade pectans, glucans, xylan, and cellulose. However, digestibility values in ruminants range between 35 and 65%, being widely accepted that the addition of fibrolytic enzymes in ruminant diets can notably increase feed conversion.

Enzymes used can be obtained from organisms able to naturally synthesize them such as fungi or bacteria [110, 111]. However, the obtained products contain an important fraction of impurities, being in many cases a mixture containing different interfering enzymatic activities. In this context, recombinant technology has been playing an important role since different fibrolytic enzymes can be produced separately using both homologous and heterologous protein expression hosts [110–115]. Some of these enzymes (β-glucanases, xylanases, mannanases, pectinases, and galactosidases) are used to specifically degrade feed components resistant to endogenous enzymes. Other enzymes, like phytases, are applied to inactivate antinutritional factors. Moreover, in some cases the supplementation with endogenous enzymes that are not produced at sufficient levels by the animal, such as proteases, lipases, and amylases are also used (Table 2). In general terms, unlike other applications previously mentioned, these enzymes are partially purified and commercialized as cellular extracts or culture supernatants that are directly used for feeding purposes, and thus commercial enzymes do not confer a single pure enzymatic activity (Table 1). However, some purification steps are required to eliminate any possible residues of genetically modified DNA and/or undesirable fermentation residues in the final product, but these purification processes are relatively simple.

**Table 2:** Marketed carbohydrases

Name	Activity	Cell factory	Animal	Company
Xylanases				
Econase XT	Xylanase	*Trichoderma reesei* (GMO)	Poultry and pigs	ABVista
Danisco xylanase	Xylanase	*Trichoderma reesei* (GMO)	Poultry and pigs	Danisco Animal Nutrition
Hostazym X	Xylanase	*Trichoderma citrinoviride* (not GMO)	Poultry and pigs	Huvepharma
Porzyme^®^9300	Xylanase	*Trichoderma longibrachiatum* (not GMO)	Poultry and pigs	Danisco Animal Nutrition
Ronozyme WX	Xylanase	*Aspergillus oryzae* (GMO)	Poultry and pigs	DSM-Novozymes
Belfeed B 1100 MP	Xylanase	*Bacillus subtilis* (GMO)	Poultry and pigs	Beldem
Xylamax™	Xylanase	NA	Poultry	BRI
Beta-glucanases				
Econase^®^GT	β-Glucanase	*Trichoderma reesei* (GMO)	Poultry and pigs	ABVista
Hostazym C	β-Glucanase	*Trichoderma citrinoviride* (not GMO)	Poultry and pigs	Huvepharma
Amylases				
Roxazyme^®^ Rumistar™	α-Amylase	*Bacillus licheniformis* (GMO)	Dairy cows	DSM-Novozymes
Multienzyme				
AvemIx^®^XG 10	Xylanase, β-glucanase	*Trichoderma reseei* (not GMO)	Poultry and pigs	Aveve Biochem
Roxazyme^®^ G2	Xylanase, β-glucanase	*Trichoderma reseei* (not GMO)	Poultry and pigs	DSM-Novozymes
Axtra^®^ XB	Xylanase, β-glucanase	*Trichoderma reesei* (GMO)	Poultry and pigs	Danisco Animal Nutrition
Axtra^®^ XAP	Xylanase, amylase, protease	*Trichoderma reesei* (GMO)	Poultry and pigs	Danisco Animal Nutrition
AvemIx^®^02 CS	Xylanase, β-glucanase, pectinase	*Trichoderma reseei* (not GMO), *Aspergillus aculeatus* (not GMO)	Poultry and pigs	Aveve Biochem
Avizyme^®^	Xylanase, amylase, protease	*Trichoderma reesei* (GMO), *Bacillus amyloliquefaciens* (GMO), *Bacillus subtilis* (GMO)	Poultry	Danisco Animal Nutrition
Endofeed	Xylanase, β-glucanase	*Aspergillus niger* (not GMO)	Poultry	GNC Bioferm
Natugrain^®^	Xylanase, β-glucanase	*Aspergillus niger* (GMO)	Poultry	BASF
Natuphos^®^ combi	Xylanase, β-glucanase, phytase	*Aspergillus niger* (GMO)	Poultry and pigs	BASF
Agal Pro BL	Alfa-galactosidase, β-glucanase	*Aspergillus niger* (not GMO), *Saccharomyces cerevisiae* (GMO)	Poultry	Biocon
Amylofeed	Xylanase, β-glucanase, amilase	*Aspergillus niger, Aspergillus oryzae* (not GMO)	Pigs	GNC Bioferm
Porzyme^®^9100	Xylanase, β-glucanase	*Trichoderma longibrachiatum* (not GMO)	Pigs	Danisco Animal Nutrition
Xybeten^®^	Xylanase, β-glucanase, cellulase	*Trichoderma longibrachiatum* (not GMO)	Poultry and pigs	Biovet
Ronozyme^®^VP	Pectinase, β-glucanase	*Aspergillus aculeatus* (not GMO)	Poultry and pigs	DSM-Novozymes
Rovabio^®^Excel	19 enzymes (xylanases, β-glucanase, and cellulases with other enzyme activities)	*Penicillium funiculosum* (not GMO)	Poultry and pigs	Adisseo
Ronozyme^®^Multigrain	Xylanase, β-glucanase	NA	Poultry and pigs	DSM-Novozymes
Ronozyme A	Amilase, β-glucanase	NA	Poultry and pigs	DSM-Novozymes
Cibenza^®^ CSM	Xylanase, β-glucanase, α-galactosidase	NA	Poultry and pigs	Novus International

Xylanases, β-glucanases and α-amylases have one declared enzymatic activity, while in some cases some secundary activities are also present in the product

*NA* information not available

Thus, although nowadays an important number of enzymes are commercially available for animal nutrition to improve animal productivity and the efficacy of utilization of natural resources, the development of optimized strategies for the production of fibrolytic enzymes is highly desirable. In this line, an extensive array of microorganisms, including bacteria (*E. coli*, *Bacillus subtilis*, and *Bacillus licheniformis*), yeast (*K. pastoris*), and fungus (*Trichoderma reesei* and *Aspergillus niger*), are being explored for the production of enzymes with interest in the feed industry. All the strategies that are being explored aim at designing fibrolytic enzymes that meet the industry requirements, which include high production yields, low production costs, easiness to scale-up, high catalytic efficiency, and improved stability under different temperature and pH conditions. This includes the use of genetic and protein engineering approaches to produce highly-active enzymes, and variants with an increased resistance to temperature and proteolysis (in many cases derived from extremophile microorganisms), ultimately resulting in a greater stability in the gastrointestinal tract. Research in this field is still underway and every year optimized processes and new products are developed and a large number of articles on this topic are published. This is particularly important considering that the global feed market is continuously growing. In particular, feed market is dominated by carbohydrases (being xylanases and beta-glucanases the most important) and phytases.

### Carbohydrases: xylanases, beta-glucanases, and amylases

Xylanases can break down xylan, which is a major polysaccharide of hemicelluloses present in plant cells and in some algae. Thus, xylanases are widely used in animal feed to degrade complex hemicelluloses. Most of the xylanases used in feed industry for enzymatic treatment of animal feed are derived from those naturally produced in fungi [116–118]. Some examples are Danisco xylanases (Danisco Animal Nutrition) and Econase XT (ABEnzymes) that are produced in *T. reesei*, whereas Prozyme 9300 (Danisco Animal Nutrition) is produced in *Trichoderma longibrachiatum*, Ronozyme WX (DSM-Novozyme) in *Aspergillus oryzae*, and Hostazym X (Huvepharma) in *Trichoderma citrinoviride* (Table 2). There is also a commercial example of a recombinant xylanase produced in bacteria (Belfeed B 1100 MP, Beldem, *B. subtilis*) (Table 2).

However, as previously described, most of these commercial products are not pure enzymes, but a complex fermentation product that in some cases contains a mixture of different enzymatic activities that may have a synergistic effect (Table 2). In many cases, xylanase is combined with β-glucanase, whereas in others amylase, protease, pectinase, phytase, and/or α-galactosidase are also present in the mixture (Table 2).

β-Glucanases are enzymes capable of breaking down cellulose and have been used in poultry, pigs, ruminants, and fish since early 1980 to facilitate the bioconversion of cellulose to animal products (Table 2). On the other hand, α-amylases are used in dairy cow nutrition to increase feed efficiency and milk production [119–123]. The most widely used is Roxazyme^®^ RumistarTM (DSM-Novozyme), which has been produced in a genetically-modified *B. licheniformis* (Table 2).

Given the importance of xylanases, β-glucanases, and α-amylases to improve the nutritional value of non-starch polysaccharides, and the increasing demand of more stable, highly-active, and non-expensive carbohydrases, different microbial hosts have been explored for their production. Although commercial carbohydrases are mainly derived from fungi, research in this field focuses in the development of bacterial and yeast-based production systems [124]. This is particularly evident for xylanases.

Considering that, in many cases, glycosylation is necessary to obtain functional and stable xylanases [125, 126], yeasts appear as the most promising heterologous expression host for their production as an alternative to fungi. Besides, some yeast have been accredited with the generally recognized as safe (GRAS) status by the American Food and Drug Administration (FDA), which brings additional value to this expression system. Altogether these advantages make yeast, and more specifically *K. pastoris*, the most widely used microorganism for xylanase production (extensively reviewed by [126]). Briefly, *K. pastoris* has been explored for the production of xylanases from *T. reesei* [127], *Aspergillus sulphureus* [128], *A. niger* [129, 130], and *Streptomyces* sp. S38 [131], among others [126]. Albeit at a lesser extent, *S. cerevisiae* has also been studied for the production of fungal xylanases [112, 132, 133]. Different enzymes in different yeast-based cell factories have been evaluated under diverse production conditions aiming to optimize enzyme production yields [133]. Li et al. and Fu et al., for instance, improved the production efficiency of the enzyme simply using an optimized sequence with the appropriate codon usage [128, 131]. On the other hand, Fang and collaborators described that *xylB* gene overproduced is not glycosylated, but it is still fully active and highly stable under different conditions [130]. Other fungal xylanases have also been shown to be non-glycosylated enzymes [118]. In line with this observation, different groups have described the production of catalytically-active eukaryotic xylanases in *E. coli* [134, 135]. Thus, *E. coli*, although to a lesser extent due to their lack of secretion system, has also been used to study different bacterial xylanases [136, 137]. Alternatively, other Gram-positive bacteria, such as *Lactobacillus* spp. and *B. subtilis*, also classified as GRAS organisms, have been used as cell factories for xylanase production purposes. Interestingly, these Gram-positive bacteria have a dual effect, since they are explored as probiotics to enhance gut health, but at the same time they are able to secrete recombinant enzymes of interest such as xylanases [138, 139]. In some cases, strategies to anchor xylanases in the bacterial cell wall have been explored [140]. Lastly, filamentous fungal expression systems (mainly *Aspergillus* spp. and *Trichoderma* spp.) have been also extensively studied for xylanase expression (reviewed in [116]) and, in fact, they are, as previously mentioned, the microorganism behind some products commercially available (Table 2). Other fungi such as *Thermoascus aurantiacus* have also been explored as potential cell factories for xylanase production [141]. Although fungi produce high levels of xylanase, they have two important limitations for industrial application, a reduced yield in fermenter conditions, and poor secretion efficiency.

### Phytases

Non-ruminant intestinal microorganisms, in contrast to what occurs with ruminal bacteria, are unable to degrade phytate from plant-derived feedstuffs [142]. This is particularly important considering that phytate is the major source of phosphorous in animal diets. Thus, traditionally, the main phosphorous source in poultry, swine, and fish diets came from the inorganic phosphates via dietary supplementation. However, the excretion of excess phosphorous by animals fed supplemented diets was accumulated in the soil and water, creating a major environmental problem [143, 144]. This challenge was minimized with the commercialization in 1991 of the first recombinant phytase, which allowed avoiding the supplementation of diets with inorganic phosphorous and, consequently, decreasing phosphorus pollution in animal waste [145].

**Fig. 1:**
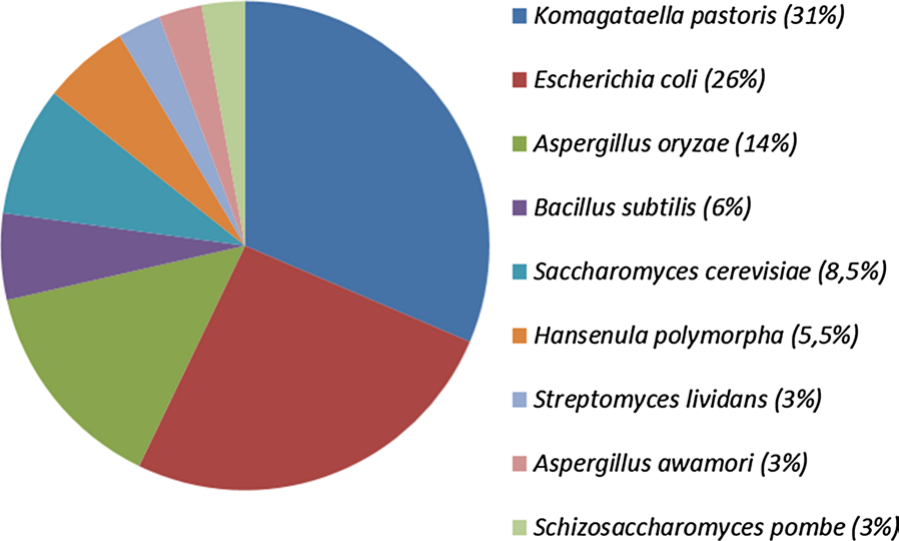
Recombinant cell factories (expressed in *percentages*) used for the production of commercial phytases

Since the development of the first commercial phytase product (Natuphos, BASF), others have been launched in the market and are nowadays available [113] (Fig. 1). Phytases that are available today are produced recombinantly in microbes including fungi (*T. resei, A. niger*, and *A. oryzae*) and yeast (*Saccharomyces pombe* and *K. pastoris*) (Fig. 1) and are widely used in diets for non-ruminant animals.

The market of feed enzymes and, more specifically of acidic phytases, has significantly grown in the last decades and its demand is estimated to continue growing in the next years. Phytases are enzymes with a large market (60% of the total feed enzyme market), which combine the capacity to improve feed efficiency with the advantage of reducing the phosphorus pollution. Due to the important phytase applicability in animal feeding, several groups are working on the design, production and characterization of phytases with optimized properties. Haefner et al., Lei et al., and Rao et al. have published extensive reviews describing all the advances in the production of phytases using recombinant cell factories [113, 142, 146].

**Fig. 2:**
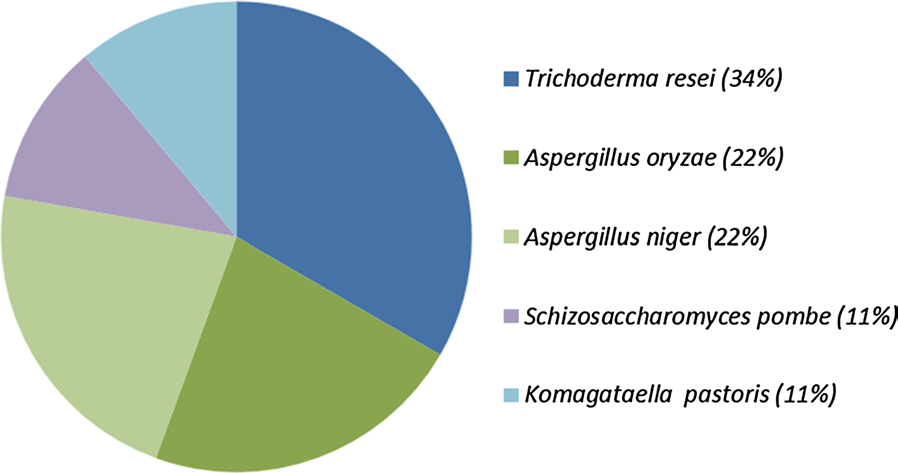
Recombinant cell factories (expressed in *percentages*) used for the production of phytases described in research articles

Research on phytase synthesis has used fungi, bacteria, yeast, and plants [113, 142, 146–148] (Fig. 2). Among fungi, the genus *Aspergillus* has been the most widely explored for the isolation of phytases with interesting properties. Besides, different species from the genus *Bacillus*, as well as some *Lactobacillus* and *E. coli* have been deeply studied [147]. Although phytases were initially isolated from their natural origin, it is widely accepted that the levels of production in such wild type strains is too low. In this context, recombinant DNA technology has allowed to make a major step toward the production of phytases at high production levels using optimized cell factories. Summarizing, among all the recombinant cell factories reported in the literature used for the production of phytases, *K. pastoris* and *E. coli* appear as the most widely used microbial factories for research purposes, whereas *A. oryzae* is the preferred option among fungi (Fig. 2).

Currently, phytase research is still focused on the identification of new phytases, but more importantly there is a clear trend towards the optimization of key properties of the already described enzymes. For the development of a new generation of phytases as feed additives, genetic and protein engineering play a key role, since they are powerful tools to develop tuned phytase variants.

Considering that the action of phytases takes place in the stomach, one of the most important requirements for phytases is a high resilience at low pHs and resistance to proteolytic degradation. Aside from this high stability to the upper digestive tract conditions, phytases used to increase animal feed efficiency must resist high temperatures to cope with the conditions of the feed pelleting process. Obviously, it is also necessary to develop enzymes with a good catalytic efficiency and produced through cost-effective production processes [113]. Aiming to produce modified phytases with optimal properties, different expression systems are being evaluated, using the sequence of phytases from different sources as starting point for further improvements. It is important to note that different expression systems (combined with phytases from different origins) produce enzymes with different biophysical and biochemical properties. For instance, the molecular mass greatly depends on the phytase origin and on the glycosylation pattern. Because bacterial phytases do not have post-translational modifications, they are easier to produce and thus have an important advantage over those from other origins. Thermostability, catalytic performance, substrate specificity, and stability at acidic pHs are greatly influenced by both heterologous system and origin. Thus far, although important advances have been achieved in this field, no enzyme with all the optimal parameters has been developed [149]. Among all those that have been studied, enzymes derived from extremophylic organisms such as *Rhizomucor pusillus*, *Thermomyces lanuginosus*, *Aspergillus fumigatus*, *Peniophora lycii*, *Agrocybe pediades*, and *Ceriporia* sp., appear as the most promising candidates [150–154]. Resolution of the crystallographic structure of some phytases allows a better understanding of this enzyme, providing a good starting point to optimize the protein engineering process. Besides, sophisticated optimization of the condition for the growth processes are also contributing to maximize titers of the variant of interest [142, 155].

In short, fibrolytic enzymes extracted from their natural sources have a low productivity yield and poor thermal and pH stability. In this context, all the efforts have focused on the development of highly active enzymes able to support extreme environments and resistant to proteases. Importantly, this needs to be done through cost-effective and high-production processes to make the new enzymes a real alternative to the existing commercial ones. In this context, bacteria and yeast represent promising alternative microbial cell factories for the production of these enzymes [124].

## Recombinant proteins for prevention and therapy

### Recombinant antibodies

The use of passive immunization (administration of antibodies) for the control of infectious disease has been recognized as a successful approach in the modern production of a wide range of animals, including pigs, cattle, sheep, goats, poultry, and fish [156]. In contrast to vaccination or active immunization, administration of immunoglobulins establishes instant immunity and provides short-term protection with no induction of immunological memory. With multifactorial infectious diseases, especially those that have proven hard to control by vaccination, the potential of passive immunization is high. Moreover, this type of therapy may be considered as an alternative to antibiotics, whose use is starting to be limited due to concerns about potential development of antibiotic-resistant bacteria.

During the last decade, the development of recombinant antibody technologies has offered the possibility for developing highly specific pathogen-specific antibodies using a cost-effectiveness and reproducible technology [157]. Some studies have demonstrated the success of using recombinant antibodies in animal production. Transmissible gastroenteritis virus (TGEV) is a positive-strand RNA virus of the family *Coronaviridae*, infecting both enteric and respiratory tissues of pigs and causing a mortality rate close to 100% when newborn pigs are infected [158]. Single-chain fragments (scFv) obtained by joining the light- and heavy-chain variable regions (VL and VH) from a monoclonal antibody (mAb) reconstitute the original VL–VH association and retain the binding specificity of the original mAb in a single polypeptide [159]. To improve the affinity of monovalent scFv, dimeric single-chain mini-antibody molecules, named minibodies or SIPs (small immunoproteins), have been generated by connecting an scFv to the dimerizing domain of immunoglobulin heavy chains. These recombinant proteins are efficiently assembled and secreted in dimeric form by mammalian cells. In vivo protection experiments on newborn piglets have demonstrated a strong reduction of virus titers in infected tissues of animals orally treated with TGEV-specific SIPs [160].

On the other hand, available vaccines for bovine herpes virus 1 (boHV-1), which causes respiratory and genital diseases in cattle, do not confer adequate protection. Koti et al. developed a bovine scFv that has a proven specificity and in vitro neutralization activity against BoHV-1 [161]. *K. pastoris* was selected over bacterial expression systems available, since yeast has protein processing and post-translational modifications similar to those present in higher-order eukaryotes as well as providing high recombinant protein yield under the influence of *AOX1* promoter. In a posterior study, the authors demonstrated that scFvs against BoHV-1 with a short linker (2 amino acids) were capable of assembly into functional multimers that conferred high avidity, resulting in increased virus neutralization in vitro compared with that of monovalent scFv [162]. These studies need to be further expanded to experiments involving virus challenges to determine the efficacy of passive protective immunity provided by bovine scFv. However, since the virus neutralization ability of the scFv in vitro was comparable to the parental mAb against BoHV-1, which reduces mortality in rabbits infected with BoHV-1, there is a future potential to be used in infected animals, to treat semen preventing the spread of BoHV-1 infection, or even by local application to treat infectious pustular vulvovaginitis caused by BoHV-1.

Another example is the foot-and-mouth disease virus (FMDV), which is a contagious viral disease that affects cloven-hoofed animals such as cattle, swine, and sheep with a potential for rapid spread. Emergency treatment by passive immunization can be used as an important control measure for FMDV outbreaks in FMDV-free regions such as the European Union. Harmsen et al. produced recombinant llama single-domain antibody fragments (VHHs) using recombinant strains of *S. cerevisiae* to confer rapid protection against FMDV by passive immunization in pigs [163].

VHHs have a number of advantages for therapeutic applications because they are well produced by microorganisms, have a high physicochemical stability and are well-suited for the construction of genetic fusions of several VHH domains [164]. Moreover, it is important to note that in that study no immunogenicity of VHHs was detected in treated pigs, which is an important aspect because passive immunotherapy can be complicated by the induction of an antibody response against the administered heterologous therapeutic recombinant antibody, especially when such antibodies are administered repeatedly.

From our knowledge, the only case of recombinant antibody produced thus far in *E. coli* as a potential therapy for animal production is related to the treatment of intrammamary infections. Bovine intrammamary infections are an important disease that causes large economical losses in the dairy industry and where passive immunization could be an interesting alternative, especially to treat infections such as those caused by *Staphylococcus aureus*, where vaccines do not confer adequate protection and the conventional antibiotic treatments have a limited success rate. Wang and collaborators constructed a recombinant scFv against fibronectin-binding protein A (FnBPA) and clumping factor A (ClfA), two important virulence factors in *S. aureus* infection [165]. However, future in vivo studies of the functionality of these scFvs are needed to confirm the potential of such scFvs.

### Other therapies

Cytokines are small molecules, which act as intercellular communication signals and play a role in various aspects of the differentiation and maturation of immune system cells and the host response to infection. Although this network is complex, there is already available information on the role of specific cytokine in the modulation of the immune system in livestock as a preventive strategy of diseases or even controlling metabolic and physiological processes. There are many in vivo studies testing targeted recombinant cytokines that stimulate the immune system to fight intramammary infections during both the lactation and the dry (the last 2 months of pregnancy, when the cow does not lactate) periods in dairy cows. Intramammary infusion of recombinant IL-2, IFNγ, IL-1β, and IL-8 in the mammary gland of lactating cows have been shown to offer protection against *S. aureus* or *E. coli* infections [166–169]. Moreover some recombinant cytokines such as IL-8 and recombinant bovine granulocyte–macrophage colony stimulating factor (rboGM-CSF) [170] have been able to foster the involution of the mammary gland during the dry period (a period where tissue of mammary gland is involuted and regenerated in the preparation for the subsequent lactation).

Dairy cows often experience decreased immune function around the time of calving, typified by impaired polymorphonuclear neutrophil (PMN) function and increased incidence of disease. Subcutaneous injections of recombinant bovine granulocyte colony-stimulating factor covalently bound to polyethylene glycol (PEG rbG-CSF) dramatically increased circulating numbers of PMN [171]. Other applications concern to the improvement of reproductive performance of production animals using IFN-τ. Recombinant buffalo IFN-τ (buIFN-τ) increased in vitro buffalo blastocyst production rate [172] although intrauterine administration of liposomized bovine IFN-τ had no effect on the length of the estrous cycle and the lifespan of the corpus luteum in dairy cows [173]. However, Shirasuna et al. found that recombinant IFN-τ was associated to greater amounts of protein, IL-8, and neutrophils in the corpus luteum of pregnant cows [174]. Lastly, supplementing recombinant porcine leukemia inhibitory factor (poLIF) in the in vitro maturation medium can improve oocyte maturation [175].

The ability of IL-3 to stimulate the development of eosinophils makes it a particularly important candidate for therapeutic use to protect against parasites. Morris et al. demonstrated that in vivo administration of poIL-3 induced a significant increase in the number of eosinophils in the blood of pigs [176]. In a similar context, chicken IFN-γ (chIFN-γ) demonstrated reductions in intracellular sporozoite development in vitro without affecting sporozoite invasion of host cells. Furthermore, chickens treated with recombinant chIFN-γ showed decreased oocyst production and significant improvement in body weight gain following an *Eimeria acervulina* challenge infection [177, 178].

All these cytokines have been produced in several recombinant systems such as mammalian cells in the case of bovine IFN-τ (boIFN-τ) [173], bovine IL-2 (boIL-2) [167], porcine IL-3 (boIL-3) [176], poLIF [175], and chIFN-γ [177]. Insect cells have been chosen for the production of chIFN-γ [177], *Brevibacillus choshinensis* for boIL-8 [168], *K. pastoris* for boIL-2, IFN-γ, and GM-CSF [170] and *E. coli* for buIFN-τ [172], boIFN-τ [174], and chIFN-γ [178].

Also in the context of the immune system modulation, the mammary serum amyloid A (M-SAA3) protein (an acute phase protein from the mammary gland) has been produced recombinantly in *E. coli* [179] and proposed as an immunostimulator of the mammary gland to fight against infections and enhance mammary involution during the dry cow period. The administration of M-SAA3 triggers an inflammatory response, the maturation of dendritic cells, and reduces the infection of mammary epithelia by pathogens such as *S. aureus*. Furthermore, relevant functions have been demonstrated in mammary function of dry cows such as the increase in neutrophil recruitment and of some key effectors of tissue involution such as metalloproteinase 9 (MMP-9) [180].

In summary, a new era of recombinant proteins, mostly key effectors in the immune system, opens the possibility to modulate physiological processes and prevent infections reducing the use of antibiotics in livestock and paving a safer and more productive future.

## Future perspectives

The use of genetic and protein engineering techniques have led to a significant progress in animal production and it is starting to have a commercial impact in this field. Nowadays it is possible to design tailor-made sequences of enzymes, which in some cases combine specific properties of different enzymes in one molecule to obtain an optimal functional protein [181]. On the other hand, this technology allows the production of recombinant hormones through cost-effective processes using microbial cells as production hosts. In addition to this, novel strategies such as those based on passive immunization are gaining ground due to the broad range of possibilities that recombinant protein production offers. In this context, although important efforts have been done toward the minimization of recombinant protein production costs, currently, much remains still to be achieved. Cost effectiveness is particularly important in the context of animal production, where marginal returns are tight. Currently, the main restriction for the application of recombinant products in animals is still the cost associated to the production processes. Overcoming this bottleneck requires developing alternative strategies to further reduce the production costs of recombinant products and there is a wide range of unexplored strategies to improve recombinant production of proteins of interest for animal production.

The use of bacterial strains with an oxidizing cytoplasm, for example, represents a good approach to improve the production yields of proteins containing disulfide bonds. In this line of work, the development and optimization of production protocols for both bacteria and yeast and the use of genetic engineering to obtain proteins with improved stability is useful. On the other hand, though yeast and bacteria are being explored as alternatives for the production of many proteins of interest, the catalogue of other promising microorganisms for this purpose is limited. Lactic acid bacteria (LAB) are an attractive alternative for recombinant protein production, since they are GRAS organisms able to produce difficult-to-express proteins [182, 183]. Even though these microorganisms have been explored in some cases for animal production purposes, especially for the production of fibrolytic enzymes, broadening their field of application would be highly convenient. They do not only show the ability to produce recombinant proteins, but they also have interesting properties as probiotics. Besides, they are able to efficiently secrete the protein of interest, which reduces the purification costs of the product of interest, and also are used for surface display purposes [184]. Interestingly, surface display, which allows to naturally anchor the enzyme of interest to the cell envelop once it is produced by the recombinant cell, has already been proven to improve the stability of an endoglucanase produced in *K. pastoris* [185].

However, it is also necessary to think beyond these classical strategies and make use of novel approaches, such as nanobiotechnology, which has been explored in other fields of research. Considering that recombinant proteins are poorly used in animal production due to their normally high associated costs, new protein formats need to be explored. Among them, inclusion bodies (IBs), which are a low-cost, highly stable, and functional protein nanoparticles mainly containing the protein of interest overproduced in a recombinant system, represent a new and appealing protein format [186, 187]. Production of recombinant proteins as IBs allows the production of any protein of interest through a much more affordable process [186–189], which could open a wide range of possibilities in animal science. Thus far, in the context of animal production, IBs from *E. coli* have only been used as a source of protein. For that, as previously described, solubilization protocols using denaturants such as urea or guanidinium chloride followed by renaturation processes have been used to obtain properly folded and functional soluble proteins [76, 190, 191]. Nevertheless, IBs have never been explored as protein-based nanoparticles for animal reproduction, enhancers of feed efficiency, or treatment purposes, with only one exception. A recent article described for the first time that IBs formed by cytokines can successfully be used as a prophylactic measure, showing that zebra fish treated with IBs are protected against a lethal infection [192]. In the same way that cytokines have been successfully produced as IBs for prevention purposes in fish, other proteins of interest (hormones, enzymes, and antibodies, among others) or other animal species could also be explored, unfolding enormous possibilities in this field. Contrarily to what has been widely believed, the formation of IBs does not only occur in *E. coli*, but in many other expression systems including yeast [193] and LAB [186, 194], meaning that their production can be conducted in a wide variety of microbial cell factories. Moreover, their size and shape are easily tunable.

Alternatively, protein encapsulation and/or coating could also be interesting nanobiotechnological approaches to increase protein stability, minimize doses and, consequently, reducing costs of proteins of interest for animal production. Nanoemulsions, liposomes, polymersomes, protein nanocapsules, polymeric nanoparticles, and hydrogel nanoparticles are some examples of different nanostructured systems used for protein encapsulation [195–198]. As an example, Diwan and collaborators described the encapsulation of gonadotropin-releasing hormone in polylactic-co-glycolic acid microspheres [199]. In another recent example, the use of gold nanoparticles has been studied to increase the stability and efficiency of a xylanase [200].

In summary, new protein formats such as IBs and encapsulation methods need to be further explored in animal science as attractive alternatives to make recombinant molecules affordable. Molecule stability and functionality can significantly be improved through these strategies.

## Conclusions

Recombinant DNA technology allows modulating protein sequence, which therefore makes it possible to obtain recombinant products with improved properties compared with those isolated from their native hosts. This has helped to make a significant step forward in the development of recombinant products for a wide array of applications, including animal production, as reviewed in the text. A broad catalogue of microbial cell factories is being explored for the successful development of enzymes, hormones, and therapeutic molecules. Nevertheless, to continue advancing in this field of study, it is necessary to make a giant leap towards the use of novel strategies that combined with recombinant technology would allow the development of products with applicability in animal science. In this context, nanotechnology, and more specifically nanostructuration, could play a crucial role in the development of a new generation of recombinant biomolecules with affordable costs for animal industry.
